# Methods for Estimating Population Density in Data-Limited Areas: Evaluating Regression and Tree-Based Models in Peru

**DOI:** 10.1371/journal.pone.0100037

**Published:** 2014-07-03

**Authors:** Weston Anderson, Seth Guikema, Ben Zaitchik, William Pan

**Affiliations:** 1 Department of Geography and Environmental Engineering, The Johns Hopkins University, Baltimore, Maryland, United States of America; 2 Department of Earth and Planetary Sciences, The Johns Hopkins University, Baltimore, Maryland, United States of America; 3 International Food Policy Research Institute, Washington, D.C., United States of America; 4 Nicholas School of Environment and Duke Global Health Institute, Duke University, Durham, North Carolina, United States of America; The University of Tokyo, Japan

## Abstract

Obtaining accurate small area estimates of population is essential for policy and health planning but is often difficult in countries with limited data. In lieu of available population data, small area estimate models draw information from previous time periods or from similar areas. This study focuses on model-based methods for estimating population when no direct samples are available in the area of interest. To explore the efficacy of tree-based models for estimating population density, we compare six different model structures including Random Forest and Bayesian Additive Regression Trees. Results demonstrate that without information from prior time periods, non-parametric tree-based models produced more accurate predictions than did conventional regression methods. Improving estimates of population density in non-sampled areas is important for regions with incomplete census data and has implications for economic, health and development policies.

## Introduction

Estimates of the distribution and growth of human population are invaluable. They are used as input to research-focused and operational applications, including emergency response, infectious disease early warning systems, resource allocation projections and food security analysis, to list only a few examples. However, obtaining reliable population estimates at the spatial resolutions required for many of these applications is a significant challenge. Census data, the primary source of population size, is often incomplete or unreliable - particularly in less-developed countries - which causes considerable problems for policy planning and decision makers. For this reason, models that can refine existing estimates of human populations or that can estimate populations in areas that lack population data altogether are of considerable importance.

Small area estimation (SAE) refers to methods for estimating small-scale characteristics of populations when there is little data, or in some cases no data at all. SAE methods are often used to produce estimates of population counts for small geographical areas, and to assess the accuracy of these estimates. Two distinct components make up SAE: design-based methods and model-based methods, which may be further divided into area-level models and unit-level models that employ either frequentist or Bayesian frameworks [1]–[2]. Unit-level models correspond to models for which information on the covariate and response variables are available for individuals, whereas area-level models only require area-averaged data for the covariates and the response. Design-based methods calculate the bias and variance of estimates from their randomization distribution induced by repeated application of the sample, while model-based methods produce inferences that are with respect to the underlying model. A significant limitation of design-based methods is that they have no means of producing predictions for areas in which no samples exist. SAE models, however, will draw information either from previous time periods or from similar areas in lieu of accurate population information [2]. This paper explores the extent to which the availability of data from previous time periods affects the choice of optimal model structure for area-level SAE models. The paper will focus on model-based methods for estimating population when no direct samples are available in the administrative unit of interest. The analysis and models discussed are limited in scope to spatial population estimation and should not be considered interchangeable with problems of temporal population prediction.

A variety of model-based methods are applicable to SAE problems, including microsimulation, areal interpolation and statistical modeling. While microsimulation and areal interpolation are valid approaches to SAE problems, this paper focuses on an assortment of statistical modeling methods. The brief review of relevant model-based estimation methods offered below is intended only to position the current research in relation to previous work on the same problem, and should not be considered a complete review of SAE as a whole.

Microsimulation produces SAE population estimates by modeling specific individuals or households and, in the case of dynamic microsimulation, life events of those individuals [3]–[4]. Spatial microsimulation builds upon static or dynamic microsimulation by explicitly representing the spatial dimension inherent in population modeling. Because the method is so computationally intensive, the simulation is often limited in its scope of application but has the unique advantage of being able to model the impact of detailed alternative policy scenarios.

In contrast to the bottom-up approach of microsimulation, areal interpolation entails distributing administrative level census data across a finer scale to produce a detailed population surface. Areal interpolation is of interest for SAE problems when coarse scale population information is available, but small-scale measurements in areas of interest are not. The most commonly used technique for producing heterogeneous population density surfaces from homogeneous zones is dasymetric mapping, which uses ancillary information to divide each zone of the source data into subzones [5]. Each subzone is assigned a population density such that the sum of population over all subzones equals the population of the original source zone [6]. In recent years areal interpolation has been used to produce gridded estimates that are more readily compatible with external modeling frameworks.

Statistical modeling in the context of SAE refers to model-based methods of producing estimates, often described as synthetic estimates, which may be used directly or blended with design-based measurements to produce a final estimate [1]–[2]. The phrase “synthetic estimate” alludes to the fact that these estimates are inferred using a model of relationships drawn from a larger domain. Synthetic estimates may be produced using either indirect implicit methods, meaning that the model assumes a homogenous relationship between dependent and independent variables across the entire small area, or indirect explicit methods, meaning that the model takes into account the spatial heterogeneity present within the small area domain. This paper analyzes both indirect implicit and indirect explicit methods, as discussed further in the following section. We emphasize that the analysis is not a complete assessment of SAE methods, but is specifically focused on methods of producing estimates when no direct estimates are available for a particular administrative unit of interest.

Many statistical models employed for SAE are regression based. One of the fundamental models is the area level model, originally employed by Fay and Herriot (1979), which takes the form
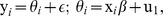


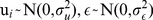
(1)where y_i_ is the direct estimator of θ_i_, x_i_ is the associated covariate for area i and β is a coefficient for fixed effects [7]. u_i_ and ε are mutually independent error terms where u_i_ represents the random effects of area characteristics not accounted for in the covariates. The best linear unbiased predictor (BLUP), 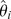, under this model is defined as
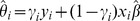
(2)where γ_i_ is a tuning coefficient defined using the variances of u_i_ and ε as 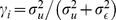. Note that Eq. 2 reduces to 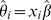 for areas without any direct samples. This model may be adapted to the unit level as proposed by Battese, Harter and Fuller (1988); however, in the present application the covariate and response variables are available at the area-level only [8].

The BLUP, 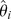, in Eq. 2 is also the Bayesian predictor under normality of the error terms when using a diffuse prior for β. When the variance σ^2^_u_ is unknown – as is often the case – it is common to replace it with a sample estimate, yielding the empirical BLUP under the frequentist approach, or the Empirical Bayes predictor under the normality assumption (the prediction then being the mean of the posterior). For area-level data, the error variance (σ^2^_ε_) must be specified from external sources. The posterior distribution of θ_i_ may alternatively be calculated by specifying prior distributions for σ^2^_u_ and β, a technique known as the Hierarchical Bayes method [1].

The linear model outlined in equation (1) may be expanded to include spatial autocorrelation to address problems that are inherently spatially dependent. These models are often used, for example, in problems of disease mapping [9]. One such model used to account for spatial autocorrelation is a linear mixed model [10]. Our analysis includes versions of both linear models and linear mixed models as established linear modeling structures against which the performance of nonlinear methods may be evaluated.

Recent studies have explored semi-parametric variations of the area-level model (equation 2), including penalized splines [11] and the M-quantile method [12]–[13], in an effort to produce a more robust inference. Models using penalized splines allow for a more flexible representation of the relationship between covariate and response variables, while an M-quantile method uses area-specific models for the regression M-quantiles of the response for estimation. A variation of the penalized splines method is included in this study, as discussed further in the following section.

The work described above demonstrates the potential of using parametric and non-parametric regression-based models to improve small area estimates. Despite this potential, the application of SAE methods in resource-poor areas is somewhat limited. In Peru, for example, although the Center for International Earth Science Information Network (CIESIN) has produced gridded estimates of population density following from statistical data, the only attempt to utilize SAE models has been in the area of poverty estimation and follows from the methods outlined in Ghosh & Rao (1994) and Rao (1999) [14]–[19]. One potential barrier to using models outlined in the SAE literature may be a lack of expertise. In our analysis we propose a number of model structures that provide accessible alternatives to more complicated methods, which often require precise user specification to produce accurate estimates. In fact, two of the models explored in our analysis – the tree-based models – require no tuning or parameterization at all, as do regression models. This makes them particularly accessible tools for providing robust model estimates.

In order to systematically identify and understand alternative model structures for population prediction in data-limited regions, we compare the predictive accuracy of six different model techniques–including both regression and tree-based methods. Each model predicts population density for districts in Peru with no available direct samples in two separate circumstances: once in which population from a previous time period is available and once in which it is not. The models use information on transportation corridors, satellite-derived land surface conditions, economic indicators and – when included – population information from the 1993 census to predict population density in 2007. Sporadic population data collection such as the gap between the 1993 and 2007 census in Peru is common in low- and middle-income countries. In the following sections we will detail the structure of the models included (Section 2), describe data sources and the required data processing (Section 3), present and discuss results (Section 4), and offer general conclusions (Section 5). The analysis is relevant for regions with limited reliable census data. In the circumstance that policy or planning scenarios require population counts as opposed to density, the area of each district can be used to transform model estimated population density back to count estimates.

## Materials and Methods

Understanding and selecting the appropriate model structure is perhaps the most important decision in the process of population modeling. The fundamental act of choosing a model structure will significantly affect the population estimate and the understanding of covariate influence. The most appropriate model structure often depends on the data available. In this analysis, six regression and tree-based models were chosen to explore how predictive accuracy and variable importance changes in the presence or absence of population information. The regression-based model structures include a linear model (LM), linear mixed model (LMM), a generalized additive model (GAM), and a multivariate adaptive regression spline (MARS) structure. The tree-based models include Random Forest (RF) and Bayesian additive regression tree (BART). A no model alternative was also included in the suite of models for reference. The strengths and weaknesses of each model structure are described in the following sections and summarized in Table 1.

**Table 1:** Summary of model structures, strengths and weaknesses.

	Model Description	Advantages	Disadvantages
**Linear Model** **(LM)**	Linear model	Simple to implement,transparent modelstructure	Unable to capture nonlinearrelationships
**Linear Mixed** **Model (LMM)**	Linear modelincorporatingspatial correlation	Explicitly accounts for spatialcorrelation, transparentmodel structure	Unable to capture nonlinearrelationships
**Generalized** **Additive Model** **(GAM)**	Non-linear extensionof a LM using asmoothing function	Able to representnonlinear relationships	Vulnerable to model over fit,which degrades predictive accuracy
**Multiple Adaptive** **Regression** **Splines (MARS)**	Penalized spline,extension of a LMusing multiple basisfunctions	Able to representnonlinear relationships	Vulnerable to model over fit,which degrades predictive accuracy
**Random Forest** **(RF)**	Baggedclassification andregression tree(CART) method	Nonparametric, designed to reducevariance and improvepredictive accuracy ofCART methods	Complex model structure, moredifficult to succinctly measurevariable importance
**Bayesian Additive** **Regression** **Tree (BART)**	Sum-of-treesmethod	Nonparametric, provides aflexible inference of therelationship between responsevariables and covariates	Complex model structure, difficultto interpret variableimportance,computationally intensive

The models were run twice: once with population density from 1993 included as a covariate, and once with it excluded, leaving only socioeconomic and environmental covariates. The two groups are intended to contrast the efficacy of model structures in the presence or absence of consistent census data. Previous population information, when included, was not modeled as a lagged effect, but rather as part of the covariate matrix. These two analyses are hereafter referred to as being with or without population data, although neither uses current period population information to estimate population density.

### Linear Models (LM)

A LM is a linear function of the form
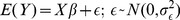
(3)where Y is the vectorized form of the response variable, X is the covariate matrix, β is a vector of coefficients and ε is a vector of the normally distributed errors [20]. In this case β may be interpreted as the relative influence of each variable. The LM model structure provides a point of comparison with the LMM to determine the marginal benefit of adding spatial correlation, and provides perspective on the performance of each of the more complex models.

### Linear Mixed Models (LMM)

A LMM is a LM in which the linear predictor may contain random effects with correlated errors [21], and takes the form:
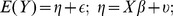


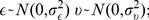
(4)where η is the linear predictor, β is a coefficient for fixed effects, X denotes the explanatory variables associated with these fixed effects and υ is a set of district-specific and possibly spatially correlated random effects that model between district variability in the response. For the purpose of population estimation in this study, errors between districts are modeled using exponential spatial autocorrelation according to the centroid of each district.

### Generalized Additive Models (GAM)

A GAM is an extension of the LM, in which the assumption of linear relationships between covariates and response variables is relaxed by replacing the linear relationship with a nonparametric smoothing function, ƒ(X), such that the form of the function becomes
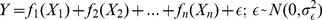
(5)


In this case a cubic spline was chosen for the nonparametric smoother with restricted degrees of freedom. In this way the GAM allows for non-linear relationships between the covariates and response variables [22]. The GAM model structure was included in this study as a relaxation of the linear features of LMs and LMMs, and as an additional non-parametric alternative to the MARS model.

### Multivariate Adaptive Regression Splines (MARS)

MARS is an extension of the linear class of models that allows for nonlinearity in the relationship between covariates and response variable by way of multiple basis functions that take the form (x−t)_+_ or (t−x)_+_ where t is a “knot point” determined in the model training process and x is the covariate. The model first enumerates basis functions to fit the data and then prunes back these functions, as would a tree-based model [23]. This gives the model the form
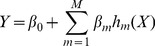
(6)where *h_m_(X)* is a basis function, or product of basis functions, and β_m_ are coefficients estimated by minimizing the sum-of-squares. The MARS model was included in this study as a variation of a penalized spline, which has been previously explored as a means of providing more robust inferences [12].

### Random Forest (RF)

Tree-based methods are often most useful for models that are highly non-linear. The most basic tree-based structure is the Classification and Regression Tree (CART), which recursively partitions the data into i subspaces and applies a very simple model to each subspace. If the loss measure used is the sum of squares, the model takes the form µ_i_ = mean(Y_i_|x_i_∈A_i_) where µ_i_ is the parameter to be predicted in subspace A_i_, Y_i_ is the set of values of the response variable on which the model is trained in that subspace and x_i_ is the matrix of the associated covariates.

One downside of CART is that the hierarchical nature of the model means relatively small changes in the data set can result in drastically different partitions within the data space, which makes drawing insight from the model structure difficult. One approach to reduce the variability inherent in predictions from CART models is to use model averaging based on bootstrapping, a method known as bagging [22]. The RF model structure is similar to a bagged CART method, except that a random subset of variables less than the total number of variables are chosen to use at the splitting point for each tree. The method originally proposed by Breiman [24] to grow B trees, each denoted by T_b_ is summarized below for a training dataset X containing M classifier variables:

1. Form bootstrap datasets x_b_ by sampling with replacement from X.
2. Select m < M variables at each node of tree T_b_. Calculate the best split for the bootstrapped dataset based on the m selected variables. Repeat this step until the specified minimum node size is reached.
3. Repeat steps 1 and 2 for each of the B trees.

Each tree is thus grown to its maximal depth. This process may be represented as:
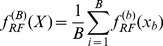
(7)


The randomization process is intended to produce uncorrelated trees (although in reality some correlation may remain) such that the aggregate result is a reduction in the variance [24]. If σ^2^ is taken to be the variance of an individual tree, and ρ is the correlation between tree predictions, then the variance may be represented as
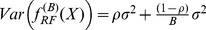
(8)


### Bayesian Additive Regression Tree (BART)

The BART model builds on regression and classification trees as a “sum-of-trees” method. The model places a prior probability on the nodes of each tree such that the tree is constrained to be a “weak learner,” biasing the tree towards a shallower, simpler structure [25]. This constraint ensures that each tree contributes only minimally to the overall fit. The model is designed to produce a flexible inference of the relationship between the sum of trees and the response variable.

Following the notation of Chipman et al. (2010), let T represent a single binary tree containing a set of interior decision nodes, terminal nodes and M = {µ_1_, µ_2_, … µ_n_} parameter values associated with each of the n terminal nodes [25]. Each decision rule is a binary split of the form {x ∈ A} vs {x ∉ A}, where A is a subset of the range of x. Each value of x, by means of binary decision nodes, is assigned to a single terminal node and therefore to a value µ_i_. for a given T and M, g(x; T, M) denotes the function that assigns µ_i_ ∈ M to x. A single tree model is therefore represented as:
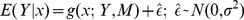
(9)Where 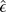 represents the normally distributed residual term centered on 0 with variance 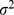. In the single tree model represented by Eq. 9, E(Y|x) equals the parameter µi assigned by g(x; T, M). Using the same notation, the sum of m trees model may therefore be expressed as:



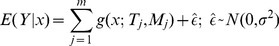
(10)Under the sum of trees model (Eq. 10), E(Y|x) equals the sum of all µ_ij_s assigned to x by the g(x; T_j_, M_j_)s. Each µ_ij_ therefore only represents part of E(Y|x) under the sum of trees model. Because each g(x; T_j_, M_j_) may be based on one or more x’s, each tree has the ability to represent either a “main effect” (single component of x, single variable tree) or an “interaction effect” (multiple components of x, multi-variable tree).

The final specification of the BART model is a prior that is imposed on all parameters in the sum of trees model (i.e. (T_1_, M_1_)…(T_m_, M_m_) and σ). The prior is designed to regularize individual tree influence such that the effect from no one tree dominates the model. The prior on T puts a larger weight on small trees and the prior on µ shrinks the fit of each terminal node proportional to the number of trees such that the contribution of any one tree decreases as number of trees increases. The full specifications of this prior may be found in Chipman et al. [25].

### Mean Model

Each of the previously described models was compared against the no-model mean alternative. The no-model mean estimate was simply calculated as the mean of available response data in the holdout dataset.

### Data

**Figure 1:**
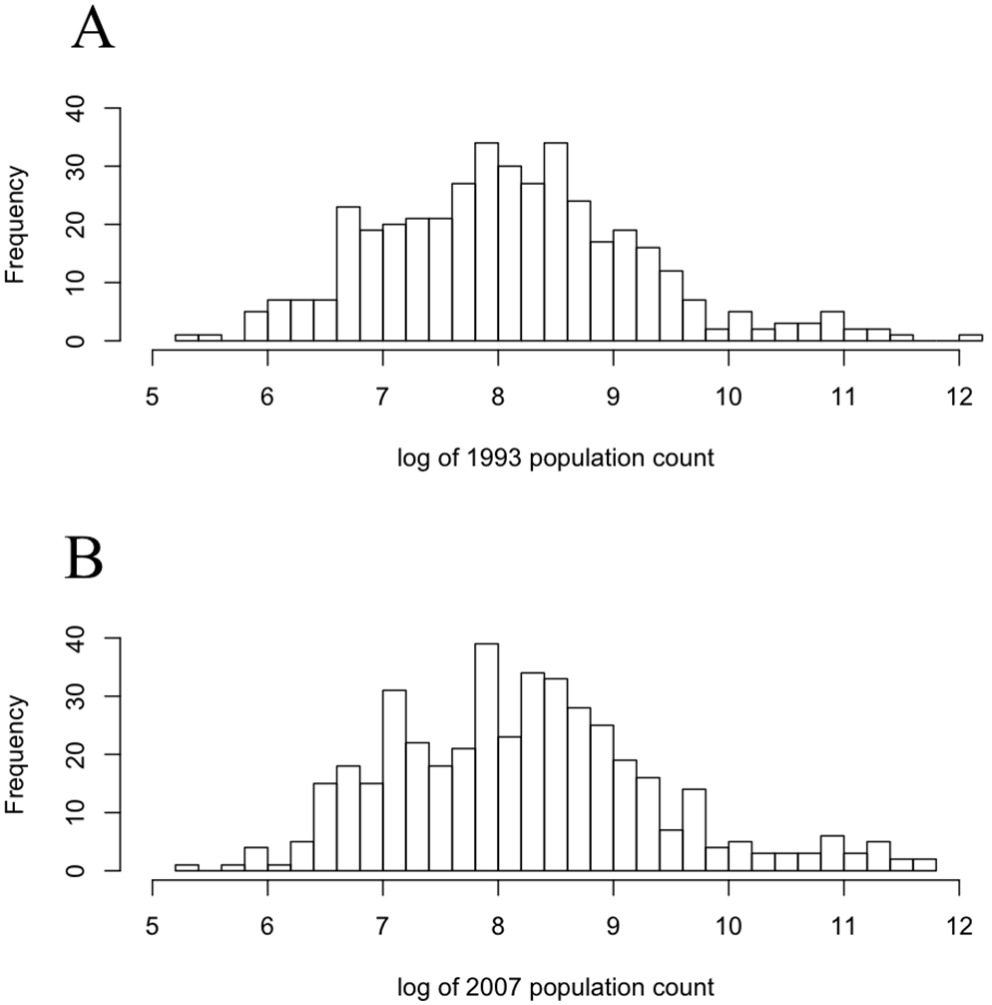
Population count by district for all regions included in the analysis for A) 1993 and B) 2007.

Peru is divided administratively into regions then provinces followed by districts. The variables used, discussed below, were calculated annually at the district level for five regions (Ayacucho, Cusco, Madre de Dios, Arequipa and Apurimac). Table 2 and Figure 1 provide descriptive statistics of the population in the five chosen regions. The total population count of all five regions increased from 1993 to 2007 (see Table 2) with a slight skew towards growth in more populous districts (see Figure 1), which broadly reflects the overall population dynamics of the country as a whole [16].

**Table 2:** Population counts for the five regions included in the analysis.

	1993 Population	2007 Population
Apurimac	381,997	404,190
Arequipa	916,806	1,112,858
Ayacucho	483,341	584,959
Cusco	1,001,898	1,154,969
Madre de Dios	67,008	102,577
Total:	2,851,050	3,359,553

Districts excluded due to irresolvable redistricting issues (see Data Consistency) are not included in the table.

**Figure 2:**
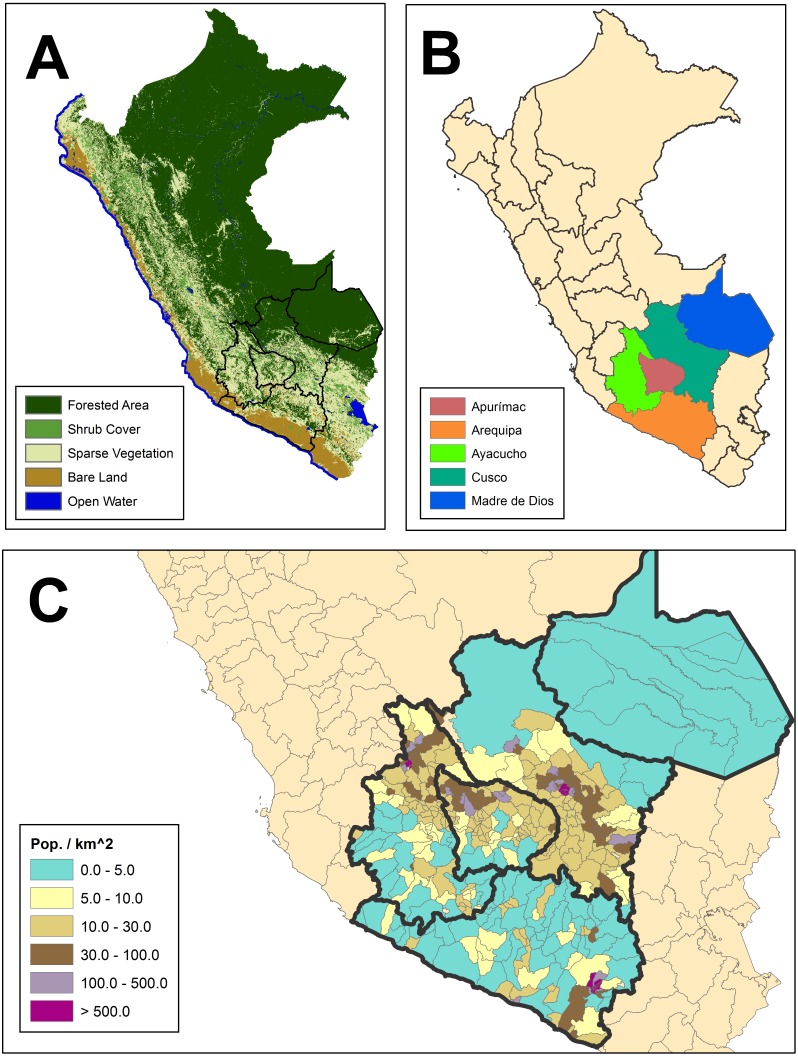
Political boundaries and topographical features of Peru. A) Classified land cover. B) Regions included in the analysis (Apurimac, Arequipa, Ayacucho, Cusco and Madre de Dios). C) Population density at the district level derived from the 2007 census.

Combined, these regions contain 42 provinces, 417 districts and span a reasonable cross section of Peruvian land cover (see Fig. 2). The country exhibits a broad range of climatic variability with land cover including rainforest, mountains and coastal areas. The five regions included in the study were chosen to be representative of Peruvian topography and to minimize redistricting within the study domain. Eight districts were created due to redistricting between 1993 and 2007, seven of which resulted from splitting an existing administrative area into two separate districts. Affected districts were recombined to pre-2007 boundaries, and all relevant variables were re-calculated.

### Response variable

Population density in 2007 was used as the response variable (Y) and was calculated using the 2007 census data downloaded from the Peruvian Institute of Statistics and Information (El Instituto Nacional de Estadística e Informática; INEI). Information on the area of each district was obtained from the GADM Global Administrative Database [26].

### Covariates

While obtaining past measures of population is a priority for improving the accuracy of population estimates, previous modeling efforts have also used a variety of ancillary variables to improve model accuracy, including information on roads, slope, nighttime lights, measures of urban areas, land use, socioeconomic characteristics and dwelling counts [27]–[28]. The scale of our study precluded the use of direct estimates of density such as counting dwellings, but measures of the land surface conditions were incorporated in the form of NDVI and daytime LST, as described in detail below. We chose to expand upon past studies that demonstrated the utility of including information on roads by selecting measures of potential transportation corridors such as rivers and inland open water. In addition to the physical characteristics of the study region, we used an index of GDP as an economic indicator for each region. In the following sections we describe the model covariates in detail and provide a brief explanation of why each was chosen for the analysis.

#### Population Density in 1993

Population density obtained from the 1993 census was incorporated as a measure of population in a previous time period. Peru conducted a census in 2005, but the methodology and results were considered flawed resulting in the 2007 census When data are available, previous population metrics have obvious value for predicting current population. The population density for 1993 was calculated similarly to that for 2007.

#### Connectivity Variables

Proximity to transportation corridors has previously proven useful in mapping population, as demonstrated by the LandScan Global Population Project, which uses the Digital Chart of the World to incorporate distance to major roads as part of a population estimation model [27]. In this study, we have extended the idea of incorporating information on transportation corridors to include navigable bodies of water. A map detailing inland roads, rivers and permanent bodies of water in Peru was downloaded from the Digital Chart of the World and aggregated to the third administrative level (districts) to obtain the density of roads, rivers and permanent bodies of water in each district. All data that originates from the Digital Chart of the World represents infrastructure circa 1992, and is static across analyses.

Density of inland roads is an indicator of urbanization and accessibility for each district. Proximity to transportation corridors–both roads and navigable rivers–may be especially important in the Amazon where large tracts of forest make transportation difficult.

Information on inland water was included because urban areas have a history of developing around bodies of water. Rivers are a valuable natural resource and provide a method of transportation. The predictive accuracy of river density may be understated in the Amazon basin because the river network is so dense that almost all land areas are accessible by river. Data for density of permanent bodies of water was categorized as a variable as well, separate from density of rivers.

#### Satellite Derived Land Surface Conditions

Remotely sensed data derived from aircraft or satellite has a fairly long tradition in population modeling. Remote sensing provides a method for detailing landscape characteristics for each district, which may in turn be linked to the population density [27], [29]–[31]. Early uses of remote sensing for population estimation were logical extensions of aerial photography, which has been used to count dwellings since the mid 1950s in areas without reliable population information [32]–[33]. Although counting dwellings becomes prohibitively time-consuming as the size of the study region increases, high-resolution remote sensing has still been used to disaggregate population counts in urban spaces under the assumption that areas with similar land cover will have similar population densities. In recent years, remote sensing has become a prominent source of environmental information, including land use and transportation patterns, which can provide valuable input to population models.

In addition to being used to disaggregate population densities, remote sensing is perhaps even more valuable for statistical population models in which it is used to estimate population density. The most common way that this is done is to relate the remotely sensed data to land use and to include that information in a regression-based model that is identified and trained using one dataset and evaluated using a separate dataset from a culturally and demographically similar area [30], [34]. While remotely sensed data are often used to derive social or economic information relevant to population density, satellite observations may also be included directly in a population model. Liu and Clarke (2002) demonstrated this by using high-resolution satellite-derived reflectance and landscape texture information to estimate population distribution within a single city [35]. The inclusion of these remotely sensed data-either directly or indirectly-allows modelers to draw insight into the underlying drivers of local population processes. For this study, remote sensing data collected by the MODIS Terra sensor was included directly as an indicator of land surface conditions. The two chosen characteristics were the Normalized Difference Vegetation Index (NDVI) and the daytime land surface temperature (LST).

NDVI measures the difference between reflectance in the near infrared and the visible spectrum. The chlorophyll in healthy vegetation strongly absorbs visible radiation while the plant cell structure reflects it. NDVI may therefore be used both as a measure of vegetative distribution and as an indicator of vegetative health. The difference in vegetative distribution may also provide information on patterns of topographical features in the landscape, as vegetative differences are often indicative of topography. NDVI was available as a 1 km resolution gridded product, but was aggregated to district averages using the administrative boundaries to match the resolution of the other covariates. Data were available as monthly composites. For this study a consistent month during the dry season was chosen (July).

Daytime LST may act to differentiate between the diverse land cover of Peru, which includes open water, bare soil, forested areas, rock and urban areas. The diurnal thermal signal of each category of land cover may provide insight into the potential habitability of that area. Daytime LST can also give an indication of the heat island effect of cities for some of the smaller districts in which impervious cover is an appreciable portion of total surface area. LST was available at 1 km resolution in 8-day composites. For this study the same composite was chosen from each year (mid July to match the NDVI). LST was aggregated to the district level using the GADM Global Administrative Areas dataset.

NDVI and LST are not perfect or comprehensive indicators of land surface conditions or specific land use. However, they are well-understood and physically meaningful variables that are indicative of differing land cover patterns and physiographic conditions that are likely to be relevant to the distribution of population. As satellite-based thematic land use classification in humid tropical regions is still a topic of active research [36]–[40], we choose to use NDVI and LST instead of relying on an error-prone land use dataset.

#### Economic Variables

While transportation networks and land cover and conditions may be important predictors of population density, they are unlikely to provide useful information for areas that have already been urbanized. A GDP index derived from the 2007 and 1993 censuses was downloaded from the INEI as an economic indicator for the analysis. Although the available GDP index is on a coarse spatial scale (provincial), it may provide significant information on interannual variability not present in other predictors.

### Data Consistency

Not all of the data from the Digital Chart of the World matched the INEI districting, although discrepancies between datasets were minor. After standardizing the data, out of 417 districts present in each year (according to the most recent INEI report) 412 districts mapped to those in the Digital Chart of the World. Districts that were omitted from the study include Jesus Nazareno, Llochegua, Huepetuhe, Majes and Kimbiri. The missing districts were due to redistricting between 1992–the year that the Digital Chart of the World was created–and the 1993 census.

### Analysis Structure

The analysis may be broadly categorized into two sections: one in which population density from 1993 is included as a covariate and one in which it is excluded, leaving only socioeconomic and environmental covariates to predict population density in 2007. The predictive accuracy of each model, both with and without population information, was evaluated using 300 repetitions of a random 10% holdout analysis. This process involves withholding 10% of the data at random (response variable and associated covariates), fitting the parameters of each model using the remaining 90% of data, and then producing predictions for the withheld 10%. The absolute difference between the prediction from each model k for each district i and the actual withheld value was calculated as mean absolute error (MAE), displayed below:
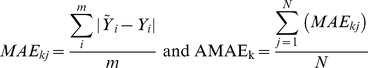
(11)


Where 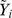 is the actual population density of district i, Y_i_ is the model prediction for district i, m is the total number of districts in the holdout dataset, MAE_kj_ is the mean absolute error of all predictions for model k in repetition j and N is the total number of repetitions. AMAE_k_ is the average mean absolute error for model k over all repetitions j. Only one year of census data was available for the response variable dataset, meaning that every prediction made in the holdout analysis was an out-of-sample prediction. Models are therefore drawing strength from the surrounding districts in that they formulate inferences based on the 90% of districts not in the holdout dataset and make predictions for the 10% of districts in the holdout sample. Such an approach is useful in many contexts but still has important limitations. Our holdout analysis cannot be generalized to countries or large regions that completely lack population data and does not test the ability of models to predict current population following calibration using old data from the same region. While calibrating each model using past data is a feasible alternative to using the information from surrounding administrative units, we were unable to test the relative strength of this methodology due to the limited temporal availability of our covariates.

The suite of models is assessed using average mean absolute error (AMAE) as a measure of general model accuracy as well as average root mean square error (ARMSE). The ARMSE penalizes large model errors more heavily than does AMAE, meaning that the difference between AMAE and ARMSE is used to assess the skill of each model in providing population density estimates for the outlier districts in the dataset. In evaluating the results of each analysis, reported levels of statistical significance are measured to a significance level of 0.05 following a Bonferroni correction for multiple pair-wise comparisons and are based on AMAE (See Tables 3 through 6). Tables 3 and 5 provide measures of model accuracy when measured using population density, while Tables 4 and 6 measure model accuracy using population count error as a proportion of actual district population to provide a more intuitive measure of model performance.

**Table 3:** Model errors and p-values assessed using density, population data included in the covariates.

	Average MAE	MAE Standard Error	LM	GAM	RF	MARS	BART	LMM
**LM**	0.051	0.033	**-**					
**GAM**	0.089	0.095	**	**-**				
**RF**	0.108	0.091	**	**-**	**-**			
**MARS**	0.051	0.036	**-**	**	**	**-**		
**BART**	0.085	0.060	**	**-**	*	**	**-**	
**LMM**	0.053	0.032	**-**	**	**	**-**	**	**-**
**Mean**	0.296	0.256	**	**	**	**	**	**

Columns 3–8 display p-values corresponding to the t-test between the MAE distributions of each row-column pair. Stars indicate statistical significance at a level of 0.01 (**) or 0.05 (*), while dashes indicate not significant.

**Table 4:** Model errors and p-values assessed using population count error as a proportion of actual district population: |Predicted–Actual|/Actual.

	Average MAE	MAE Standard Error	LM	GAM	RF	MARS	BART	LMM
**LM**	0.105	0.036	-					
**GAM**	0.108	0.042	-	-				
**RF**	0.150	0.054	**	**	-			
**MARS**	0.109	0.036	-	-	**	-		
**BART**	0.133	0.049	**	**	**	**	-	
**LMM**	0.104	0.036	-	-	**	-	**	-
**Mean**	0.491	0.207	**	**	**	**	**	**

Population data included in the covariates. Stars indicate statistical significance at a level of 0.01 (**) or 0.05 (*), while dashes indicate not significant.

**Table 5:** Model errors and p-values assessed using density, population data not included in the covariates.

	Average MAE	MAE Standard Error	LM	GAM	RF	MARS	BART	LMM
**LM**	0.371	0.123	**-**					
**GAM**	0.412	0.121	**	**-**				
**RF**	0.207	0.121	**	**	**-**			
**MARS**	0.371	0.174	**-**	**	**	**-**		
**BART**	0.298	0.157	**	**	**	**	**-**	
**LMM**	0.302	0.124	**	**	**	**	**-**	**-**
**Mean**	0.296	0.256	*	**	**	**-**	**-**	**-**

Columns 3–8 display p-values corresponding to the t-test between the MAE distributions of each row-column pair. Stars indicate statistical significance at a level of 0.01 (**) or 0.05 (*), while dashes indicate not significant.

**Table 6:** Model errors and p-values assessed using population count error as a proportion of actual district population: |Predicted – Actual|/Actual.

	Average MAE	MAE Standard Error	LM	GAM	RF	MARS	BART	LMM
**LM**	0.515	0.118	-					
**GAM**	0.496	0.116	-	-				
**RF**	0.408	0.106	**	**	-			
**MARS**	0.479	0.129	-	-	**	-		
**BART**	0.436	0.110	**	**	-	**	-	
**LMM**	0.453	0.125	**	**	**	-	-	-
**Mean**	0.491	0.207	-	-	**	-	**	-

Population data not included in the covariates. Stars indicate statistical significance at a level of 0.01 (**) or 0.05 (*), while dashes indicate not significant.

The diversity of model structures included in the analysis required the use of multiple measures of variable influence in the analysis of the results. The relative importance of each variable in the LM and LMM was measured using the β coefficient from the final fitted model. In this case the β coefficient indicates the linear relationship between covariate and response variable. Variable influence in the MARS model was based on the contribution of a variable towards reducing the model’s generalized cross-validation (GCV) score. GCV is an approximation of the leave-one-out cross-validation using a squared error loss measure [22]. The measure of variable importance used for a GAM was the increase in average MSE that results from removing a specific variable. Variable importance in the RF model was evaluated using two separate indices. The first is based on perturbing each variable and recording the effect on the out-of-bag accuracy as measured by average MSE, while the second measures the decrease in node impurities- measured by the residual sum of squares- that results from splitting on a variable. Variable importance in the BART model was evaluated by the number of times a variable was used as a splitting decision in a tree, averaged over all trees. Due to the discrepancy between measures of variable influence, direct comparisons between models cannot be made. Instead, the shift in relative variable influence between analyses is explored within each model to understand how each model is affected by the presence of population density information in the covariates.

The analysis was conducted using R, with the following packages and functions: the linear model was fit using the glm() function from the stats package with a Gaussian link function and variable selection conducted using the step() command; the generalized additive model was fit using the gam() function from the mgcv package with added penalty terms for each new variable using the select command; the linear mixed model was fit using the glmmPQL() function from the MASS package with exponential spatial correlation and a Gaussian link function; the random forest model was fit using the function randomForest() from the package of the same name; the multivariate adaptive regression splines model was fit using the earth() function from the package of the same name; and the Bayesian Additive Regression Tree was fit using the bart() function from the BayesTree library. No additional specifications were required for the RF, MARS or BART models.

## Results and Discussion

Despite the fact that the regression based models (LM, LMM, GAM and MARS) provided the most skilled predictions of population density when 1993 population density was included in the covariates, these models- with the exception of the LMM- provided among the worst predictions when no population information was included (see Tables 3 and 5). The inclusion of spatial correlation (represented by the LMM) produced the best predictive accuracy among regression models, but the model still performed no better than the no-model alternative (Table 5). Although there are minor differences in model performance when assessed using population counts, notably the improved performance of GAM, the relative model accuracy remains unchanged (see Tables 4 and 6). This result indicates that when population information was not available regression-based models (both parametric and non-parametric) were unable to capture the relationship between indicator variables and current population density.

In contrast to the regression models, the RF model – a non-parametric tree-based model - provided among the most skilled estimates when 1993 population density was not included in the covariates, but among the least skilled estimates when it was (see Tables 3 through 6). Notably, when population information was not included the RF model was the only model to significantly outperform the no-model alternative as assessed by population density. When measured using population counts instead of density, both tree-based models (RF and BART) significantly outperform the no-model alternative. The shift in relative model performance indicates that the relationship between previous population and current population at a district scale can be modeled effectively using regression methods, but the relationships between ancillary variables and population require a more flexible model structure better able to handle nonlinearity and high variance. This discrepancy in predictive accuracy demonstrates the potential of tree-based models for estimating population in data-limited areas.

The differences in model accuracy as evaluated by ARMSE as opposed to AMAE are minimal in terms of ordinal rank but entail consistently larger mean error estimates with increased standard errors. The systematic difference in model accuracy as measured by AMAE and ARMSE (results not shown) implies that the model estimates contained disproportionately large errors in a small number of predicted districts. Although an expanded dataset containing a greater number of districts may help to reduce the standard error of the model estimates across models, in data-scarce regions it’s very likely that the data available to train models is limited.

The covariate influence of all models was explored to understand the differences in variable importance between the two analyses. Although the most direct measure of variable importance is model dependent, which precludes direct comparisons between measures of variable importance, relative comparisons between analyses are instructive. When population density from 1993 was included in the covariates, LM, LMM and MARS – the three models that provided the best population density estimates for the analysis– all indicated that previous population density was the most significant variable as assessed by their respective measures of variable importance (Table 7). This relative variable importance is not surprising, but is an important point of comparison for evaluating the models that do not include 1993 population density information in the covariates.

**Table 7:** Measures of variable importance, population data included in the covariates.

	Previous Popdensity	Roads	River Water	X coordinate	Y coordinate	NDVI	LST Day	GDP	Perm Water
LM Beta Values*	0.979	-	–0.0199	-	-	-	-	-	-
LMM Beta Values*	0.979	0.0198	-	NA	NA	-	-	-	-
**GAM Percent reduction in MSE**	505.22	–0.92	–0.87	–0.07	1.6	1.78	-	-	-
MARS GCV*	100	5	-	-	-	-	-	-	-
**RF Percent reduction in MSE**	22.19	1	2.26	1.35	3.9	3.33	3.74	2.15	1.21
**RF Inc. Node Purity**	204.94	81.85	22.21	16.25	27.27	19.65	18.34	3.11	0
**BART Mean number of splits**	68.28	19.89	13.84	11.95	10.76	11.71	31.21	17.88	27.29

Stars indicate the models producing the most accurate estimates. Dashes indicate variables that were discarded by the model during variable selection.

When 1993 population information was not included in the analysis, nearly all of the models indicated a greater number of covariates were significant, many of which the models had previously excluded (see Tables 7 and 8). Random Forest – the model that provided the best population density estimates for the analysis - indicated that the majority of remaining covariates had comparable variable importance (Table 8). The flexibility of the RF model structure compensated for a lack of previous population density information more effectively than regression-based models by incorporating information from the available covariates. The second tree-based method, BART, similarly produced estimates that were more accurate than all regression-based models (both parametric and non-parametric), although not statistically distinct from the mean model.

**Table 8:** Measures of variable importance, population data not included in the covariates.

	Previous PopDensity	Roads	River Water	X coordinate	Y coordinate	NDVI	LST Day	GDP	Perm Water
**LM Beta Values**	NA	0.154	–0.184	0.117	–0.102	-	-	-	-
**LMM Beta Values**	NA	0.166	-	NA	NA	-	-	-	-
**GAM Percent reduction in MSE**	NA	–4.37	–1.3	-	–2.33	–2.59	-	-	-
**MARS GCV**	NA	100	45.9	15.8	26	22.2	16.8	-	-
RF Percent reduction in MSE*	NA	3.19	-0.91	2.45	4.7	4.88	5.61	4.33	-0.63
RF Inc. Node Purity*	NA	116.79	45.36	35.23	47.67	32.61	41.14	7.78	0.18
**BART Mean number of splits**	NA	55.03	28.43	18.09	33.01	30.33	39.62	37.19	26.61

Stars indicate the models producing the most accurate estimates. Dashes indicate variables that were discarded by the model during variable selection.

**Figure 3:**
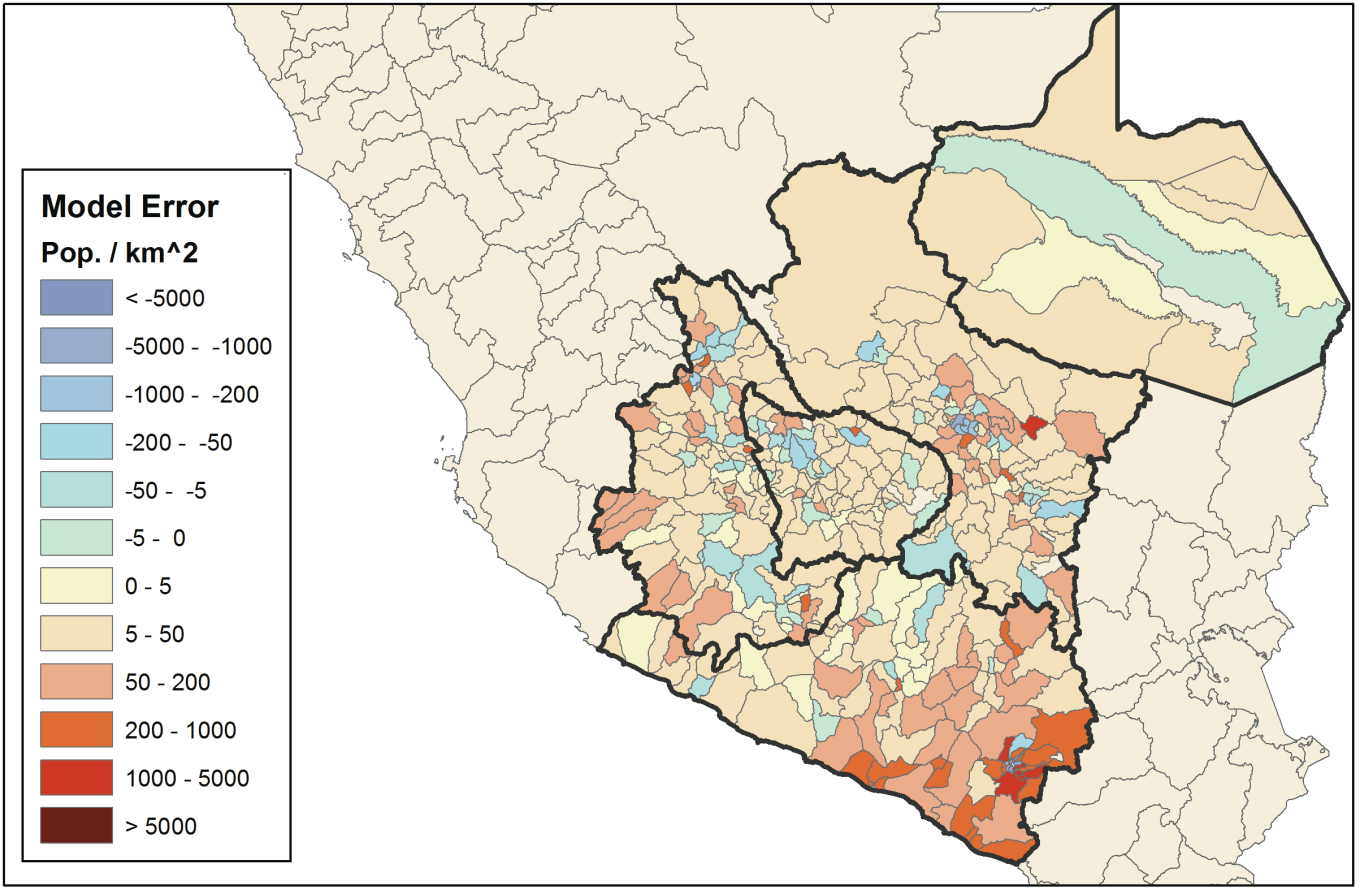
Random Forest model error by district.

**Figure 4:**
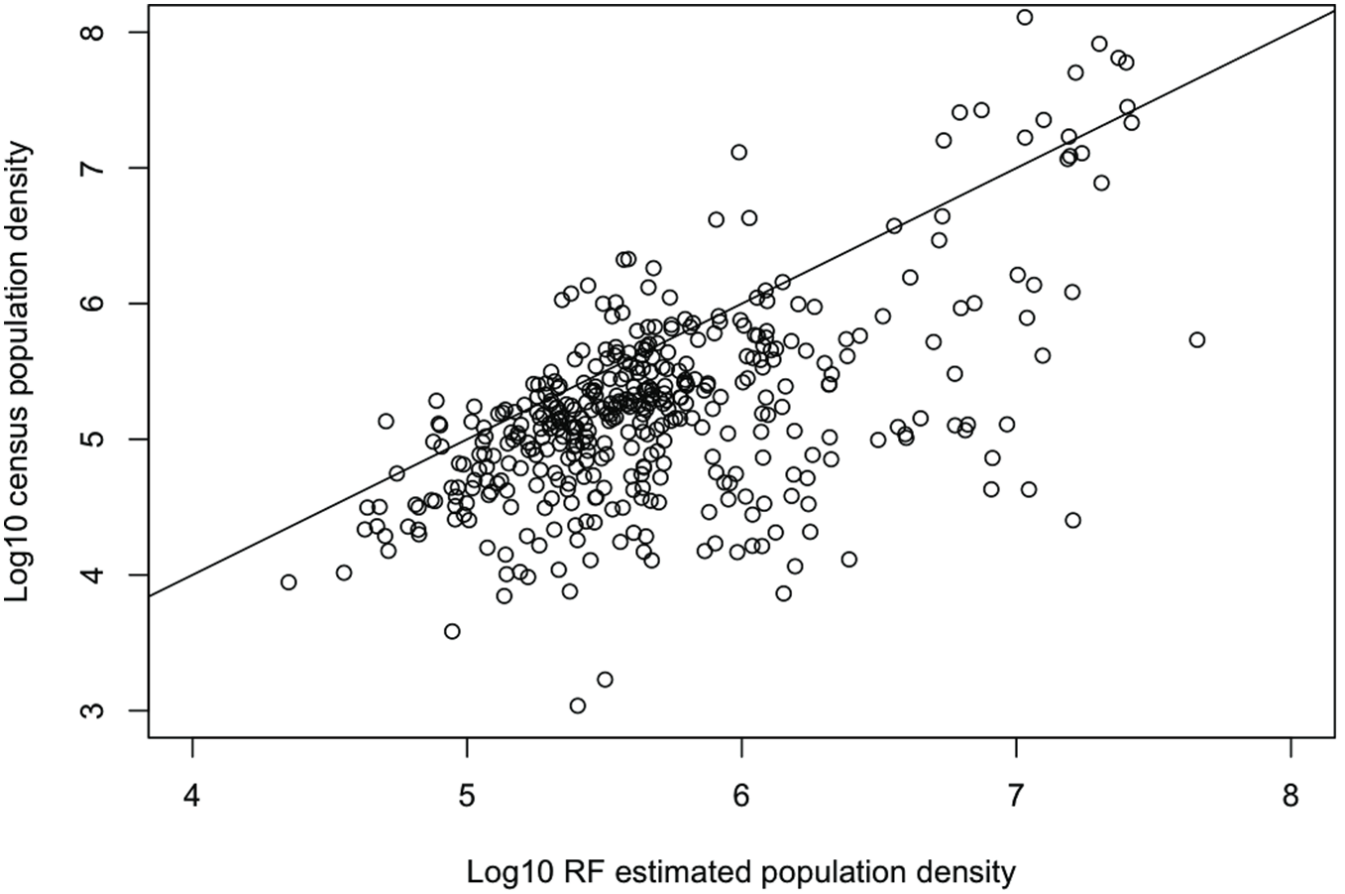
Census population density vs. Random Forest estimated density. 1∶1 line plotted for reference.

Random Forest population density estimates and model errors are explored spatially and in their relation to actual district population density to better understand the performance of the model. Figure 3 demonstrates some spatial dependencies in the model errors, particularly in the southeast, although performance was mixed across much of the study domain. Isolated districts across the domain display large model errors, which demonstrates the limitations of using model-based population estimates alone. The population dynamics in these districts are likely controlled by variables not captured in our analysis or exhibit an anomalous relationship between the covariates and response variable. This serves as a point of caution when interpreting any single prediction from a model optimized for predictive skill across a large and diverse study region. Figure 4 shows that RF tended to overestimate population density in general but slightly underestimated the population density of the most dense ∼10% of districts. The overestimation bias for lower-density districts is not surprising given the relatively small margin available for underestimation in such districts. The inability of the RF model to produce accurate population density estimates for the most population dense districts implies that the resolution of the analysis – which in this case is the district level – may have been insufficient to capture the upper extreme of population-density due to heterogeneity of the response variable within each district. Dense urban areas may account for the majority of a district’s population but a relatively minimal proportion of its land area, on which many covariates were based.

**Figure 5:**
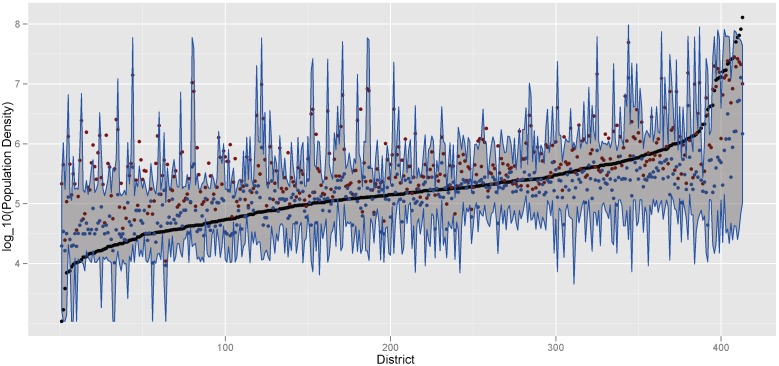
Random Forest uncertainty analysis. Observations of district population density (black points) are ordered from lowest to highest density. The Random Forest mean (red point), median (blue point) and interval between the 5^th^/95^th^ percentiles (blue lines) illustrate the uncertainty in each corresponding model estimate.

To explore the uncertainty in estimates made using the Random Forest model, a Quantile Regression Forest (QRF) was used to characterize the distribution around each model estimate [41]. Just as the RF model is used to estimate the conditional mean of a response variable, E(Y |X = x), the QRF model is used to estimate quantiles in the conditional distribution of a response variable as E(1{Y ≤y}|X = x). Figure 5 depicts the actual district population densities, ordered from smallest to largest, the RF mean estimate for each district and the QRF estimate of the 5^th^, 50^th^ and 95^th^ percentiles. The RF mean is shown to overestimate density in all districts with the exception of the ∼10% of districts with the highest population density. Although nearly all of the population density observations fall within the bounds defined by the 5% and 95% QRFs, these bounds are often quite wide, demonstrating the uncertainty inherent in predicting population density without prior information on population.

Figure 5 demonstrates that the RF mean is significantly skewed towards the high end of the QRF distribution, at times falling outside of the 95^th^ percentile. Such behavior is an indicator that the RF model is producing some trees in the ensemble with significantly higher population density estimates than the rest of the ensemble. This is likely another indicator that, as previously discussed, the data is highly nonlinear and may not be well represented in some of the area-level covariates due to heterogeneity within individual districts. The QRF median (50^th^ percentile) was included in Figure 5 as an illustration of a potential alternative to the RF mean for problems that demonstrate such nonlinearities. While the QRF median provides a more stable estimate that is more accurate for all but the highest density districts, it also significantly reduces the range of the estimates. Therefore, while the median may be preferable in this particular model, it is still logical to begin with the RF mean as an estimate in most situations.

## Conclusions

For regions in which data limitations preclude the use of reliable demographic information, it is important that model structures effectively incorporate all available data. Producing reliable small area estimates of population density for areas that lack direct samples is a problem of interest for resource allocation, disease early warning, and food security analysis. Such estimates are vital for decision makers operating in regions limited by incomplete or unreliable census data. It is often these same data-limited regions that lack information from previous time periods for use in training and testing models.

The improvement in predictive accuracy demonstrated by the RF model represents practical value for decision makers and policy makers. The average MAE of MARS, GAM and LM are twice as large as for RF, and that of LMM is one and a half times as large. Even when compared to the average model in which population information is available, the RF model produces errors that are only two to four times greater and therefore still useful in an applications context. While the improvements to predictive accuracy are limited to the case in which no previous population information is available, it is in just such cases that model estimates must be relied upon most heavily.

The results of this study illustrate that for non-sampled areas a regression-based model may not be the most effective model structure depending on the continuity and consistency of available census data. Without information from prior time periods, the flexibility provided by the non-parametric tree-based models produced more accurate predictions than did conventional parametric and non-parametric regression methods. The predictive accuracy of tree-based non-parametric models in population modeling is an area that has been largely unexplored, but which warrants further study as a flexible alternative to conventional regression based methods.
